# Feeding cessation alters host morphology and bacterial communities in the ascidian *Pseudodistoma crucigaster*

**DOI:** 10.1186/s12983-016-0134-4

**Published:** 2016-01-14

**Authors:** Susanna López-Legentil, Xavier Turon, Patrick M. Erwin

**Affiliations:** Department of Biology & Marine Biology, and Center for Marine Science, University of North Carolina Wilmington, 5600 Marvin K. Moss Lane, 28409 Wilmington, USA; Center for Advanced Studies of Blanes (CEAB-CSIC), Accés Cala S. Francesc 14, 17300 Blanes Girona, Spain

**Keywords:** Tunicate, Bacterial diversity, Symbiosis, Filter-feeding, Mediterranean sea, Next-generation sequencing, 16S rRNA, TEM, SEM

## Abstract

**Background:**

Ascidians can associate with abundant and diverse consortia of microbial symbionts, yet these communities remain unexamined for the majority of host ascidians and little is known about host-symbiont interactions.

**Methods:**

We coupled electron microscopy and 16S rRNA gene tag pyrosequencing to investigate the bacterial communities associated with the colonial ascidian *Pseudodistoma crucigaster*, a species endemic to the Mediterranean Sea that has a life cycle with two phases: actively-filtering (active) and non-filtering (resting) forms.

**Results:**

Resting colonies exhibited a reduced branchial sac (feeding apparatus) and a thickened cuticle. Electron microscope images also suggested higher abundance of colonizing microorganisms on surfaces of resting colonies. Accordingly, bacterial sequences associated with environmental sources (sediment and biofilms, >99 % similarity) were detected exclusively in resting colonies. Bacterial communities of *P. crucigaster* colonies (active and resting) were dominated by 3 core taxa affiliated (>94 % similarity) with previously described symbiotic Alphaproteobacteria in marine invertebrates. Shifts in rare bacteria were detected when ascidians entered the resting phase, including the appearance of strictly anaerobic lineages and nitrifying bacterial guilds.

**Conclusions:**

These findings suggest that physical (thickened cuticle) and metabolic (feeding cessation) changes in host ascidians have cascading effects on associated bacteria, where modified oxygen concentrations and chemical substrates for microbial metabolism may create anaerobic microhabitats and promote colonization by environmental microorganisms.

**Electronic supplementary material:**

The online version of this article (doi:10.1186/s12983-016-0134-4) contains supplementary material, which is available to authorized users.

## Background

Symbiosis is a close interaction between two or more species that has permitted some species to overcome their physiological limitations by exploiting the capabilities of others, thus playing significant roles in the evolution of plants and animals [1–4]. As with all animals, marine invertebrates are known to form a wide range of symbiotic associations with other organisms. Perhaps most common are associations with Bacteria [5–12] and Archaea [13–16], forming what is known as a holobiont [17]. The nature of these associations ranges from loose relationships (e.g. opportunistic epibionts) to obligate symbioses, depending on each host species and microbial symbiont [18–23]. These diverse symbiont communities can participate in the production of defensive secondary metabolites [24–27], provide UV-absorbing molecules [28] or enhance host metabolism through microbial processes such as photosynthesis [29], sulfate reduction [30] and nitrification [15].

Ascidians or sea-squirts (Chordata, Ascidiacea) are a diverse group of sessile marine invertebrates characterized by their secreted gelatinous or leathery tunic [31] composed of tunicine (a cellulose-like polysaccharide). Because these animals are sessile as adults and cannot escape their predators, ascidians are well-known as prolific producers of defensive secondary metabolites [32–34], several of which function as chemical protection against predation, spatial competition or colonization by fouling or pathogenic microorganisms [25, 35–38]. In addition to their ecological impact, these bioactive secondary metabolites have substantial importance for biotechnology and drug discovery, as many exhibit novel pharmaceutical applications especially as anti-cancer drugs [39]. To date, few studies have aimed to pinpoint the origin of these secondary metabolites and have uncovered molecules of both bacterial symbiont [40, 41] and animal host origin [42, 43].

Indeed, few ascidians have been examined for the presence of microbial associations, with most studies focusing in tropical colonial species. Didemnid tropical ascidians are well-known to establish symbiotic relationships with unicellular cyanobacteria, including the genera *Prochloron*, *Synechocystis* and *Acaryochloris* [16, 19, 44–50]. More recently, the widespread occurrence of diverse bacterial (e.g. Proteobacteria*,* Bacteroidetes) and archaeal lineages (e.g. Thaumarchaeota) has also been demonstrated in the inner tunic of these tropical animals [16]. Temperate ascidians are more frequently associated with bacteria from phyla other than cyanobacteria. The polycitorid *Cystodytes dellechiajei* and the botryllids *B. schlosseri* and *B. violaceus* are known to be associated with mostly Proteobacteria from the Alpha and Gamma classes and members of the phylum Bacteroidetes [43, 51]. The solitary styelid *Molgula manhattensis* is colonized by a spiroplasma-like bacterium that is also found in the gonads [51], while the perophorid *Ecteinascidia turbinata* (found in both tropical and temperate waters) is associated with intracellular bacteria from the class Gammaproteobacteria that are believed to produce the anti-tumoral compound T-743 [52, 53].

The colonial ascidian *Pseudodistoma crucigaster* Gail, 1972 (Pseudodistomidae), is an endemic colonial ascidian from the Mediterranean Sea [54–56]. In the western Mediterranean, this species is commonly found between 5 and 20 meters depth and attached to rocky surfaces occupied by photophilic communities [54, 57]. *P. crucigaster* is also characterized by morphological polymorphism, with at least three chromatic varieties described: yellow, grey and orange [54, 56]. The life cycle of this species exhibits a marked seasonality [55, 57]. Gonads appear in winter months and incubating embryos can be found between January and July, when mature larvae are released, followed by a period of reproductive inactivity. Growth occurs also during winter-spring, with a decrease in size of the colonies at the beginning of summer [57]. During summer, a non-feeding or resting form is observed in many colonies, characterized by sealed siphonal apertures, the development of a thick, glossy cuticle over the colonies and a regression in area of the colonies [55, 57, 58]. After a short period (<4 weeks), colonies shed the glossy cuticle and resume filtering and growth [57].

The main aim of this study was to characterize the microbial community associated with actively-filtering (active) and non-filtering (resting) colonies of the Mediterranean ascidian *Pseudodistoma crucigaster.* We hypothesized that the morphological and metabolic changes between active and resting forms would impact the structure of associated microbial communities. To address these aims, we coupled 16S rRNA gene tag pyrosequencing and electron microscopy techniques to characterize the bacterial community structure and composition, visualize bacterial morphotypes and determine major structural changes in the tunic of filtering and non-filtering colonies.

## Results

### Structure of the tunic and resting zooids

**Fig. 1:**
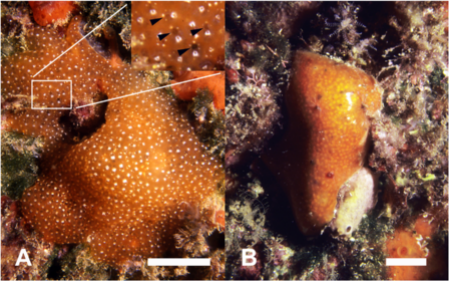
Underwater images of the colonial ascidian *Pseudodistoma crucigaster*. **a** Actively filtering form (*active*) and **b** resting form. Arrowheads point to some siphonal apertures. Scale bar represent **a** 2 cm and **b** 1 cm

**Fig. 2:**
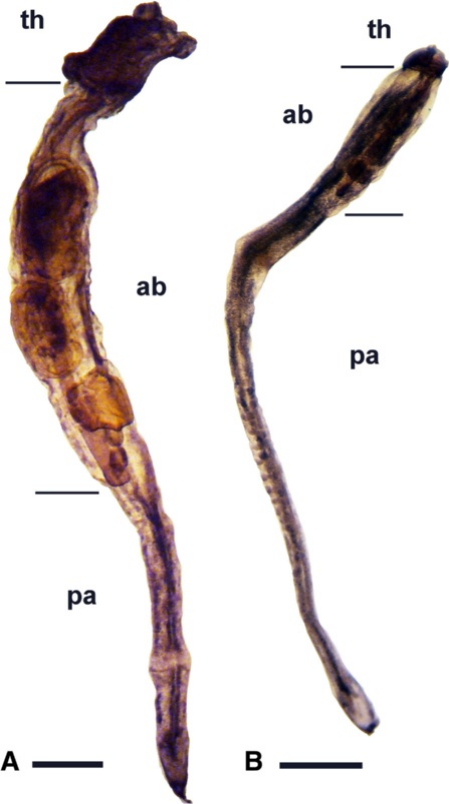
Light microscopy images of a filtering (**a**) and resting (**b**) zooid of *Pseudodistoma crucigaster*. See the strong regression of the thorax (branchial sac) and the accumulation of reserves in the abdomen. th: thorax; ab: abdomen; pa: postabdomen. Scale bars represent 1 mm

**Fig. 3:**
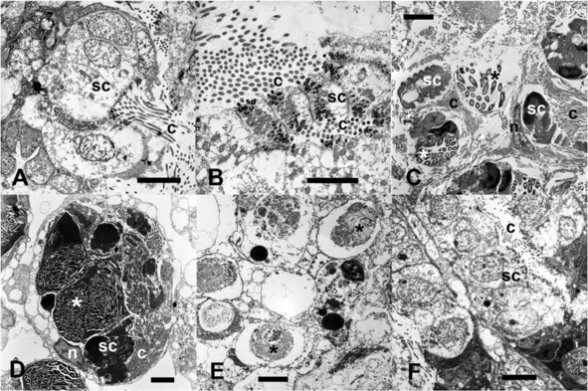
Transmission electron micrographs of the branchial sac of active and resting zooids. **a** Stigmatal cells (sc) of an active phase, with cilia (c) in the lumen of the stigma. **b** Active phase, tangential section of distal part of stigmatal cells (sc); cilia (c) can be seen at both sides. **c** Resting form showing branchial sac filled with macrophages containing degenerating stigmatal cells (sc) with cilia (c) still recognizable, and apoptotic material (asterisks); (n): nucleus of a macrophage cell. **d** Resting phase, close-up of a macrophage with nucleus (n) and cytoplasm filled with big phagosomes, some with unrecognizable apoptotic material (asterisk), some with condensed stigmatal cells (sc) with their ciliary bundles (c). **e** Final resting phase in which cells with big vacuoles (some of them with cytoplasmic remains, asterisks) fill the thorax. **f** Beginning of the regeneration, new stigmatal cells (sc) are formed, and cilia (c) are visible in the newly formed lumen of the stigma. Scale bars for images represent 5 μm (A to F) and 15 μm (C)

The non-feeding (resting) form of *P. crucigaster* was easily discerned macroscopically, as the siphonal apertures (Fig. 1a) were sealed and a glossy pellicle covered the colonies (Fig. 1b). Internally, the zooids were characterized by a strong regression of the branchial sac and the accumulation of reserves in the abdomen and postabdomen (Fig. 2). At the ultrastructural level, active forms were characterized by a functional branchial sac with cilia in the lumen of the stigmata (Fig. 3a-b). In resting forms, the main feature observed was the proliferation of macrophages containing degenerating stigmatal cells in the inner lumen of the branchial sac (Fig. 3c-d). By the end of the regression process, the branchial sac was completely reabsorbed and cells with big vacuoles (some of them with cytoplasmic remains) filled the thorax (Fig. 3e) before the onset of the regeneration of the branchial sac structures (Fig. 3f). The gut tissues did not undergo lysis and their integrity was maintained during the whole process. The development of a new functional thorax, the shedding of the cuticle, and the reopening of the siphons on the colony surface marked the end of the resting period. No mortality associated with passage through a resting form was detected in previous monitoring efforts [57].

**Fig. 4:**
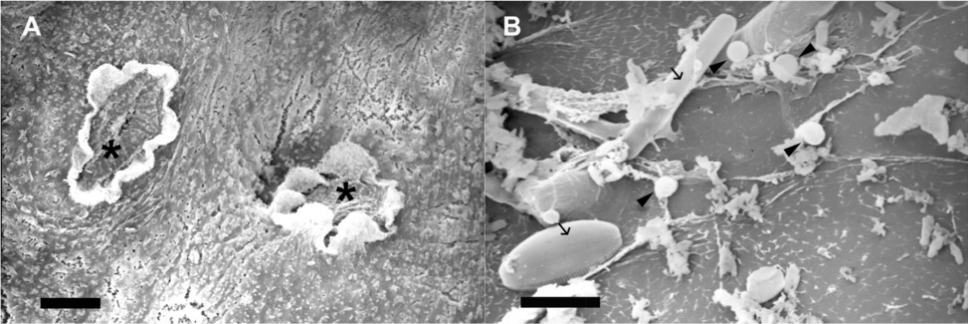
Scanning electron micrographs of the colony surface of the ascidian *Pseudodistoma crugicagster.* Photos show **a** the surface of an active colony with two siphonal apertures (asterisks), and **b** the surface of a non-filtering colony featuring some microorganisms (arrows point to diatoms, arrowheads indicate some bacteria). Scale bars represent **a** 0.2 mm and **b** 10 μm

**Fig. 5:**
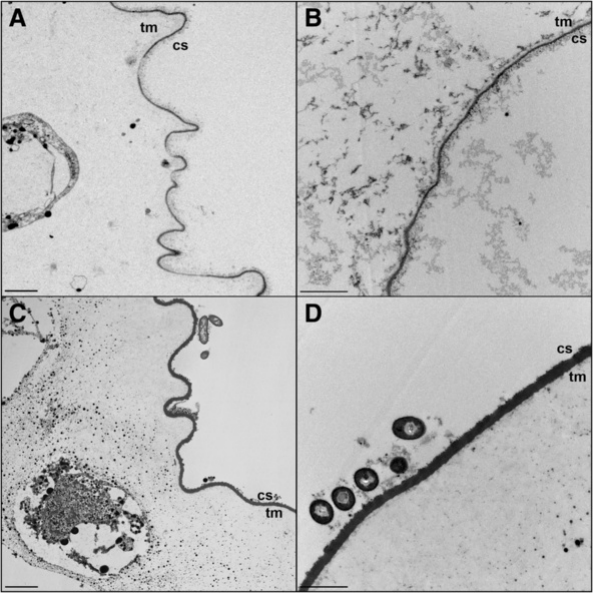
Transmission electron micrographs of the tunic of the colonial ascidian *Pseudodistoma crucigaster*. **a** Actively-filtering colony and **b** detail of its cuticle. **c** Resting or non-feeding colony, **d** showing a thicker cuticle and different bacterial morphotypes attached to it. Tunic matrix (tm) and cuticle surface (cs). Scale bars represent 1 μm

Electron microscope observations also showed open siphonal apertures in the active colonies and clean surfaces devoid of epibionts (Fig. 4a, Fig. 5a-b), while the surface of resting colonies often appeared fouled by different microorganisms, including diatoms and bacteria (Fig. 4b, Fig. 5c-d). In addition, the cuticle of the active form of *P. crucigaster* was much thinner (0.05-0.08 μm, Fig. 5a-b) that the one observed for the resting colonies (0.15-0.2 μm, Fig. 5c-d). Further TEM observations revealed few bacteria inside the tunic of both active and resting forms of this species, while in resting colonies several bacteria were observed attached or in close proximity to the outside cuticle and in direct contact with the seawater (Fig. 5d).

### Structure of the microbial communities

**Fig. 6:**
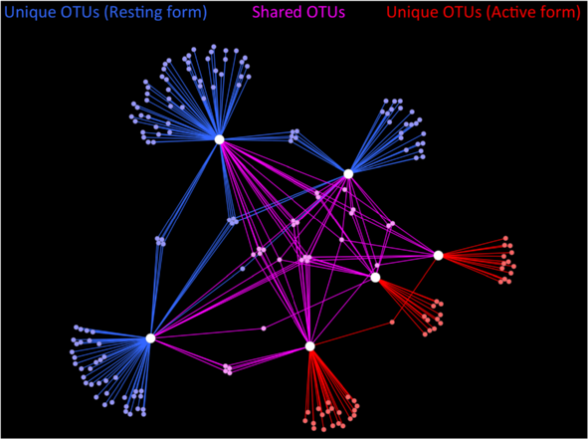
Network of bacterial OTUs in *Pseudodistoma crucigaster*. OTUs present in active colonies are in red, resting colonies in blue or both in pink. Symbiont OTUs (small, colored nodes) and host individuals (large, white nodes) are shown, with edges coded by specificity

Microbial communities in resting forms of *P. crucigaster* exhibited significantly higher richness than those in active forms, for both observed and expected richness metrics (Table 1). Network analysis indicated that these additional OTUs in resting forms included both OTUs specific to one individual host and several shared among hosts (Fig. 6). Comparisons of microbial community evenness revealed higher values in active hosts, although this difference was only significant in one of two tests (Table 1). No differences in diversity metrics (Inverse Simpson, Shannon Index) were observed between symbiont communities of resting and active hosts (Table 1).

**Table 1:** Metrics comparing the richness, evenness and diversity of bacterial symbiont communities

Diversity metric	Measure	Active	Resting	*P*-value
Richness	Observed OTUs (S_obs_)	28 ± 5	62 ± 10	<0.05*
	Expected OTUs (S_Chao1_)	33 ± 6	87 ± 19	<0.05*
Evenness	Simpson Index (E_1/D_)	0.10 ± 0.02	0.05 ± 0.01	0.076
	Smith & Wilson (E_var_)	0.48 ± 0.02	0.38 ± 0.02	<0.05*
Diversity	Inverse Simpson (1/D)	3.2 ± 1.2	2.9 ± 0.1	0.819
	Shannon Index (H)	1.4 ± 0.4	1.9 ± 0.1	0.294

Statistical comparisons (Student’s t-test) between communities in active and resting colonies of the ascidian *P. crucigaster* are shown, with significant values indicated with asterisks (*)

Community-level statistical analyses revealed higher similarity in symbiont communities from resting ascidians (62 %) compared to active colonies (39 %) and clustering of resting colony symbionts but not of symbionts from active colonies. No significant differences in the structure (PERMANOVA, *P* = 0.19) and heterogeneity (PERMDISP, *P* = 0.10) of microbial symbiont communities were detected between active and resting ascidian colonies based on the relative abundance of symbiont OTUs. However, significant differences were detected in the phylogenetic structure of microbial communities between active and resting ascidians (U = 0.853, *P* < 0.001; W = 0.441, *P* < 0.001, Additional file 1: Figure S1), indicating differences in the relatedness of symbiont OTUs among hosts.

### Microbial diversity and composition

**Fig. 7:**
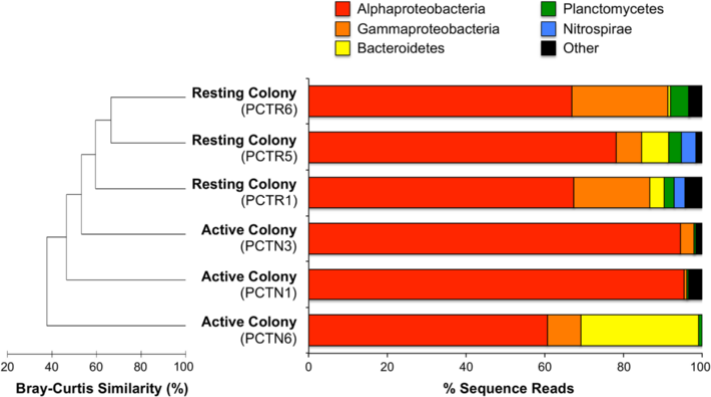
Microbial community similarity and composition in active and resting colonies of *Pseudodistoma crucigaster*. Dendrogram (*left*) show on Bray-Curtis similarity values between ascidian samples and bar charts (*right*) show the relative abundance of bacterial phyla in each host

A total of 196 microbial symbiont OTUs were recovered from active and resting colonies of *P. crucigaster*, representing ten bacterial phyla and one archaeal phylum. Representatives of six phyla were detected in both active and resting colonies (Proteobacteria, Bacteroidetes, Planctomycetes, Verrucomicrobia, Actinobacteria), with an additional four bacterial phyla (Nitrospirae, Firmicutes, Cyanobacteria, Acidobacteria) and the single archaeal phylum (Thaumarchaeota) detected only in resting colonies (Fig. 7). OTUs affiliated with Proteobacteria dominated the communities of both active and resting ascidian colonies, with the sub-class Alphaproteobacteria comprising for the majority (61–95 %) of all symbiont sequences.

A small portion of microbial OTUs (13 %, *n* = 26) was shared between active and resting colonies (i.e., present ≥1 individual of each form, Fig. 6), yet these OTUs accounted for the vast majority of symbiont sequences recovered (73–98 %). In fact, two OTUs (001, 002) within the Alphaproteobacteria (order Rhodobacterales) dominated symbiont communities in both active and resting hosts, alone accounting for 53–92 % of all sequences. Together with a third, related OTU (006, Alphaproteobacteria, Rhodobacterales), these 3 core OTUs were the only taxa consistently recovered from both active and resting forms (i.e., present in all host individuals). Notably, these OTUs were distinct from previously described sequences (<96 % similarity; Table 2) and matched most closely to symbiont-derived sequences for other marine invertebrates, including sponges and octocorals (Table 2). Of the remaining 22 shared OTUs, only one (OTU-023) was present in all active colony individuals and some (2 of 3) resting colonies. This OTU was closely related to the 3 core OTUs (Alphaproteobacteria, Rhodobacterales) and matched most closely to an octocoral-associated sequence (94 % similarity). On the contrary, the only two OTUs (003, 004) present in all resting hosts and some (1 or 2) active colonies were classified as Gammaproteobacteria or Bacteroidetes and exhibited high similarity (>97 %) to sequences from environmental sources (sediment, Table 2).

**Table 2:** Relative abundance, taxonomic classification and closely BLASTn match of top bacterial OTUs

	% Sequence reads								
OTU	*N1*	*N3*	*N6*	*R1*	*R5*	*R6*	Phylum (Class)	Lowest Taxon	Top BLAST match
001	85	43	28	55	58	59	Proteobacteria (α)	Family Rhodobacteraceae	Sponge-associated (94.8 % EU350873)
002	7	44	25	3	14	5	Proteobacteria (α)	Order Rhodobacterales	Octocoral-associated (95.7 % DQ395396)
003	*-*	3	5	6	<1	5	Proteobacteria (γ)	Order Alteromonadales	Deep sea sediment (98.1 % AB694145)
004	*-*	*-*	18	2	<1	<1	Bacteroidetes	Family Flammeovirgaceae	Deep sea sediment (97.5 % FN562913)
005	*-*	*-*	*-*	*-*	*-*	17	Proteobacteria (γ)	Class Gammaproteobacteria	Coral-associated (100 % KC527310)
006	2	5	<1	1	1	<1	Proteobacteria (α)	Order Rhodobacterales	Sponge-associated (94.8 % EU350873)
007	*-*	*-*	*-*	9	<1	*-*	Proteobacteria (γ)	Family Xanthomonadaceae	Crab-associated (99.5 % AB476258)
008	3	<1	*-*	<1	*-*	<1	Proteobacteria	Phylum Proteobacteria	Ascidian-associated (97.2 % KF799034)
009	*-*	*-*	7	<1	*-*	*-*	Bacteroidetes	Genus Roseivirga	Ascidian-associated (99.5 % KF798800)
010	*-*	*-*	2	2	*-*	<1	Proteobacteria (α)	Class Alphaproteobacteria	Ascidian-associated (100 % KF798861)**
011	*-*	*-*	*-*	<1	3	2	Planctomycetes	Order Phycisphaerales	Biofilm (100 %, JF272039)**
012	*-*	*-*	<1	*-*	5	*-*	Bacteroidetes	Genus Maribacter	Octocoral-associated (100 % KF181050)**
013	*-*	*-*	<1	<1	*-*	2	Planctomycetes	Order Phycisphaerales	Microbial mat (96.7 % JN435867)
014	*-*	*-*	*-*	2	<1	*-*	Nitrospirae	Family Nitrospiraceae	Sediment (100 % AJ704710)**
015	*-*	*-*	*-*	1	1	<1	Nitrospirae	Family Nitrospiraceae	Seawater (100 % KC706459)**

Results are shown for bacteria associated with active (N) and resting (R) forms of *P. crucigaster*

The majority (87 %) of OTUs detected in *P. crucigaster* where exclusive to active colonies (*n* = 45) or resting colonies (*n* = 125) and accounted for 2–27 % of symbiont communities (Fig. 6). Of the 45 OTUs exclusive to active hosts, 17 were singletons (occurring as a single read in the dataset) and none were present in all host individuals (Fig. 6). Of the 125 OTUs exclusive to resting hosts, 60 were singletons and 4 OTUs were preset in all hosts (OTU-011, -015, -022, -062; Fig. 6). Similar to OTU-003 and -004, these 4 OTUs were classified to various bacterial taxa (Planctomycetes, Nitrospirae, Betaproteobacteria, and Alphaproteobacteria, respectively) and exhibited near identical (>99 %) matches to sequences from environmental sources (Table 2).

## Discussion

The colonial ascidian *Pseudodistoma crucigaster* has a complex life cycle that includes a growth phase in winter, when the colony is actively filtering (active), and a reduction in size in summer, when non-filtering (resting) forms are common [55, 59]. Electron microscope observations revealed that active colonies were characterized by a thin cuticle lacking the minute protrusions present in some ascidian families [60]. The cuticle of resting colonies was much thicker and had a glossy appearance in live colonies. Although formal quantitative analyses were not conducted, the external surfaces of resting colonies were also consistently colonized by epibionts (bacteria and diatoms). Internally, the major feature observed was a regression of the branchial sac during the resting period followed by a regeneration of the thorax before the colony reassumed its filtering activity. This process was very similar to what has been described for the didemnid ascidian *Polysyncraton lacazei* [61], for which a brief resting period lasting a few weeks was accompanied by the appearance of a glossy cuticle and a full thorax renewal at the end. In *P. lacazei*, this process was interpreted as an asexual rejuvenation phenomenon to regenerate the parts of the zooids that had the highest metabolic activity (branchial sacs), and is different from the ‘survival budding’ described by Nakauchi [62]. Both *P. lacazei* and *P. crucigaster* non-feeding colonies were also observed in summer, corresponding with a period of food-shortage for filter-feeders in the Mediterranean Sea [63, 64].

Next-generation sequencing of partial 16S rRNA genes revealed a stable core bacterial community associated with active and resting forms of *P. crucigaster* with marked differences in the rare microbiome. Microbial communities in the tunic of both active and resting colonies of *P. crucigaster* were dominated by 3 core taxa affiliated with Alphaproteobacteria and related to symbiont sequences from other marine invertebrates. These results are consistent with a growing body of literature indicating that Proteobacteria often represent the most common bacterial lineages in association with ascidians [16, 43, 50, 51, 65–67]. No additional symbiont OTUs were shared between active colonies, with the remaining bacterial groups occurring sporadically among individual hosts. In contrast, a broader diversity of bacterial OTUs (e.g., Planctomycetes and Nitrospirae) were present in all resting colonies and absent from active colonies. Further, these symbiont groups matched closely (>99 % similarity) to environmental sequences previously detected in seawater, sediment and biofilm communities. These results indicate that a small group of core symbionts dominate active and resting colonies of *P. crucigaster*, while pumping cessation leads to changes in the rare microorganisms that grow in and on ascidian colonies, possibly by bacterial colonization from ambient environmental sources.

Notably, several symbiont taxa identified in the resting form of *P. crucigaster* were affiliated with phylogenetic lineages that have specific functional capabilities, providing insight into the putative functionality of the ascidian holobiont. These included representatives of the family Nitrosomonadaceae, a lineage of Betaproteobacteria capable of the first step in the nitrification pathway (ammonia oxidation, [68]), and of the phylum Nitrospirae, a gram-negative bacterial lineage capable of the second step of nitrification (nitrite oxidation, [69, 70]). The presence of these functional guilds indicates that aerobic nitrification is taking place in the tunic or cuticle surface of *P. crucigaster*. Previous research on the ascidian microbiome indicates that ammonia-oxidizing symbionts are widespread in ascidian hosts, though comprised of archaeal lineages [16]. For example, in the colonial ascidian *Cystodytes dellechiajei*, no evidence for bacterial-mediated nitrification was observed while ammonia-oxidizing Archaea and their corresponding ammonia monooxygenase genes were detected in the tunic of this Mediterranean species [71]. Since these functional guilds were only detected in resting colonies, the disruption of filter-feeding in *P. crucigaster* may also impact symbiont community functioning and metabolism. Further research is needed to test this hypothesis and investigate microbiome functionality in ascidian hosts, including the metabolism of the dominant, unique Rhodobacteraceae symbiont identified herein.

Morphological and metabolic changes in resting ascidian hosts may also impact associated bacterial communities by altering oxygen conditions and waste products utilized for microbial metabolism. The observed formation of a thick cuticle in resting ascidian colonies may limit oxygen diffusion or alter oxygen dynamics in the tunic, leading to anaerobic microhabitats within some sections of the ascidian body. For example, a strictly anaerobic class of Chloroflexi (Anaerolineae) was detected in resting colonies and absent from active colonies, indicating that microhabitats supporting anaerobic metabolism may exist in resting colonies of *P. crucigaster*. In addition, some bacterial taxa may obtain substrates for metabolism from the ascidian hosts (e.g. ammonia-rich waste products) and be deprived of these when the animal stops feeding. Changes in the metabolic activity of resting colonies, triggered by the interruption of filter feeding in summer [63], could have cascading effects on symbiont metabolism. Thus, appearance of new microhabitat conditions and decreases in available substrates for metabolism may potentially contribute to the observed changes in the bacterial communities between active and resting colonies of the Mediterranean ascidian *P. crucigaster*.

As with many sessile invertebrates, colonial ascidians are known to produce secondary metabolites to avoid predation and surface colonization by other organisms [36, 37, 72, 73]. Members of the genus *Pseudodistoma* are known to be rich in cytotoxic alkaloids, amino alcohols, tryptophan-related compounds, alkyl amines and nucleosides [74–81]. In particular, *P. crucigaster* produces amino alcohols with strong antimicrobial activity [75, 81]. These compounds were obtained after extraction of the full organism; therefore, it is unknown whether the animal, a microbial symbiont or both are responsible of their synthesis. Although the origin of these compounds is not yet resolved, our microscopic observations suggest that resting colonies of *P. crucigaster* lacked the anti-fouling activity featured by active colonies, results corroborated by the presence of environmental and biofilm-associated bacteria in DNA sequence data. The persistence of dominant symbionts across active and resting *P. crucigaster* colonies suggest that these core symbionts may not be involved in the synthesis of chemical defenses against potential epibionts. More likely, the reduction in host metabolism from feeding cessation results in limited production of antimicrobial compounds and the observed colonization of the ascidian body by environmental bacteria. Characterization and comparisons of microbial communities in active and resting ascidian hosts provides indirect insight into the origins of chemical defenses and direct insight into discerning transient and persistent members of the ascidian microbiome.

## Conclusion

We found that the bacterial communities associated with the colonial ascidian *P. crucigaster* were dominated by a few unique symbionts and exhibited shifts in rare taxa when the animal host ceased filter-feeding and entered a resting phase. Microscopy observations indicate this phase is characterized by branchial sac renewal, the appearance of thick cuticle and a likely colonization of the tunic surface by environmental bacteria, whereas active colonies maintained a thin cuticle and a surface mostly devoid of epibionts. Sequence data support these findings, with the appearance of environmental bacteria associated exclusively with resting ascidian colonies, possibly resulting from lower production of anti-fouling chemicals due to reduced host metabolism. In addition, strictly anaerobic lineages and nitrifying bacteria were detected only in resting forms of *P. crucigaster*, suggesting that morphological and metabolic changes may also impact associated bacteria by altering oxygen concentrations and chemical substrates for microbial metabolism. The unique life cycles of ascidians provide ideal study systems for linking changes in host metabolism, chemical defenses and physical microhabitats to shifts in the structure and function of associated bacterial communities, providing insight into host-symbiont interactions and potential control mechanisms of holobiont metabolism.

## Methods

### Sample collection and preservation

Colonies of the orange morph of the Mediterranean ascidian *Pseudodistoma crucigaster* (Fig. 1) were collected by SCUBA diving between 10 and 15 m depth and at least 2 m apart from each other in Tossa de Mar (41°43’13.62”N, 2°56’26.90”E; NE Spain). Samples were collected September 6, 2012 and included three actively-filtering (active) colonies (Fig. 1a) and 3 non-feeding (resting) colonies (Fig. 1b). A few zooids from each colony were carefully dissected under a stereomicroscope and observed under light microscopy. Samples for genetic analyses were preserved in absolute ethanol and stored at -20 °C until analyzed. Samples for scanning electron microscopy (SEM) were fixed in a solution of 4 % formaldehyde and stored at room temperature until processed. Samples for transmission electron microscopy (TEM) were fixed in a solution of 2.5 % glutaraldehyde and 2 % paraformaldehyde buffered with filtered seawater, incubated overnight at 4 °C, rinsed at least three times with filtered seawater and stored at 4 °C until processed.

### Electron microscopy

For SEM analyses, the upper part of each colony was dissected under a stereomicroscope, deposited on a stub covered with bi-adhesive tape, critical-point dried and sputter-coated with gold. Observations were made using a Cambridge H-120 microscope. For TEM analyses, small pieces (ca. 3 mm^3^) of the tunic and zooids of active and non-feeding colonies were observed on a JEOL JEM-1010 (Tokyo Japan) coupled with a Bioscan 972 camera (Gatan, Germany). Both SEM and TEM observations were made at the Microscopy Unit from the Scientific and Technical Services of the University of Barcelona.

### DNA extraction, PCR amplification and amplicon pyrosequencing

Tunic samples (ca. 2 mm^3^) from three actively-filtering to three resting colonies of *P. crucigaster* were dissected with a sterile scalpel, including the surface cuticle and excluding zooids, and extracted using the DNeasy Blood and Tissue kit (Qiagen®) according to the manufacturer’s instructions. PCR amplification of a partial 16S rRNA gene fragments and barcoded amplicon sequencing were performed at Molecular Research, LP (Shallowater, TX), using bacterial Tag-Encoded FLX Amplicon Pyrosequencing with the forward primer 926 F 5′-AAA CTY AAA KGA ATT GAC GG-3′ and reverse primer 1392R 5′-ACG GGC GGT GTG TRC-3′ [82].

### Data processing and statistical analysis

Sequence depth ranged from 712 to 1,612 reads per sample (average = 1,050 reads, ±150 SE) and did not differ significantly between the three active and three resting forms of *P. crucigaster* (t-test, *P* = 0.37). Raw sequence data were processed in the software package mothur [83] using a previously described bioinformatics pipeline [16, 66] that included stringent de-noising, quality filtering and removal of non-target and putatively chimeric sequences (see Additional file 2: Table S1 for details). High quality sequences were grouped into operational taxonomic units (OTUs) based on 97 % sequence similarity (average neighbor cluster algorithm, [84]), following alignment to the Silva database (v. 102) and trimming to an overlapping alignment space (293 bp). Each sequence read was assignment to taxonomic groups (Greengenes taxonomy template, [85]) and the taxonomic assignment of each OTU was constructed by majority consensus [84]. Raw sequence data were deposited as flowgrams (sff files) in the Sequence Read Archive of the National Center for Biotechnology Information (SRA NCBI) under the accession number [GenBank: SRA272795].

Bray Curtis similarity matrices were conducted on relative OTU abundance per individual host and visualized in cluster plots. Permutational multivariate analyses of variance (PERMANOVA) and permutation analyses of dispersion (PERMDISP) were conducted to compare the structure and heterogeneity of bacterial communities between active and resting colonies. All statistical analyses were performed in Primer v. 6 (Plymouth Marine Laboratory, United Kingdom; [86, 87]). In addition, beta-diversity metrics based on the phylogenetic structure of microbial communities in active and resting colonies were computed using weighted and unweighted unique fraction (UniFrac) algorithms [88], as implemented in mothur [83] with significance determined by Monte Carlo simulations. OTU networks were created to visualize the overlap in bacterial OTUs between and among active and resting ascidian hosts using Cytoscape 2.8.3 [89], with edge and nodes files created manually from relative abundance OTU tables and an edge-weighted, spring-embedded algorithm used to construct the network.

## Additional files

Additional file 1: Figure S1. Multi-dimensional scaling plots based on (A) unweighted and (B) weighted Unifrac distances among bacterial communities in active (red triangles) and resting (blue triangles) colonies of the colonial ascidian *Pseudodistoma crucigaster*. (DOCX 112 kb) Additional file 2: Table S1. Bioinformatic pipeline for 454 sequence read processing in mothur (v.1.29.2). Commands (in bold), input file types and settings are shown. (DOCX 112 kb)
