# Supplementary material for: Feeding cessation alters host morphology and bacterial communities in the ascidian Pseudodistoma crucigaster
Source: Front Zool. 2016 Jan 14;13:2. doi: 10.1186/s12983-016-0134-4 (PMC4712478; doi:10.1186/s12983-016-0134-4)
Supplement: Additional file 1: Figure S1. — Multi-dimensional scaling plots based on (A) unweighted and (B) weighted Unifrac distances among bacterial communities in active (red triangles) and resting (blue triangles) colonies of the colonial ascidian Pseudodistoma crucigaster. (DOCX 112 kb) [file 12983_2016_134_MOESM1_ESM.docx]

**Figure S1**. Multi-dimensional scaling plots based on (A) unweighted and (B) weighted Unifrac distances among bacterial communities in active (red triangles) and resting (blue triangles) colonies of the colonial ascidian *Pseudodistoma crucigaster*.
