# Supplementary material for: Feeding cessation alters host morphology and bacterial communities in the ascidian Pseudodistoma crucigaster
Source: Front Zool. 2016 Jan 14;13:2. doi: 10.1186/s12983-016-0134-4 (PMC4712478; doi:10.1186/s12983-016-0134-4)
Supplement: Additional file 2: Table S1. — Bioinformatic pipeline for 454 sequence read processing in mothur (v.1.29.2). Commands (in bold), input file types and settings are shown. (DOCX 112 kb) [file 12983_2016_134_MOESM2_ESM.docx]

SUPPLEMENTARY MATERIAL

**Table S1.** **Bioinformatic pipeline for 454 sequence read processing in mothur (v.1.29.2).** Commands (in bold), input file types and settings are shown.

| **Command** | **Input File(s)** | **Settings** |
| --- | --- | --- |
| trim.seqs | fasta, oligos, qfile | maxambig=0, maxhomop=8, bdiffs=0, pdiffs=1, qwindowaverage=35, qwindowsize=50, minlength=200 |
| get.seqs | fasta, group, accnos* | n.a. |
| unique.seqs | fasta | n.a. |
| align.seqs | fasta, reference | processors=4 |
| screen.seqs | fasta, name, group | start=28465, optimize=end, criteria=75 |
| filter.seqs | fasta | vertical=T, trump=. |
| degap.seqs** | fasta | n.a. |
| unique.seqs** | fasta, name | n.a. |
| align.seqs** | fasta, reference | n.a. |
| filter.seqs** | fasta | vertical=T, trump=. |
| classify.seqs | fasta, name, template, taxonomy | cutoff=60 |
| dist.seqs | fasta | output=lt, cutoff=0.25 |
| cluster | phylip, name | cutoff=0.25 |
| classify.otu | list, name, taxonomy | label=0.03 |
| make.shared | list, group | label=0.03 |
| get.oturep | phylip, list, fasta | label=0.03 |
| unifrac.weighted | tree, group | distance=lt, random=t |
| unifrac.unweighted | tree, group | distance=lt, random=t |
|  |  |  |

*Metaxa (v1.1) used to create the accnos file (see methods)

**These commands represent an alignment issue fix (de-gapping and re-alignment required to avoid misalignment of identical sequences resulting from different sequence lengths during initial alignment).
